# Epigenetic Features of HIV-Induced T-Cell Exhaustion Persist Despite Early Antiretroviral Therapy

**DOI:** 10.3389/fimmu.2021.647688

**Published:** 2021-06-04

**Authors:** Genevieve E. Martin, Debattama R. Sen, Matthew Pace, Nicola Robinson, Jodi Meyerowitz, Emily Adland, John P. Thornhill, Mathew Jones, Ane Ogbe, Lucia Parolini, Natalia Olejniczak, Panagiota Zacharopoulou, Helen Brown, Christian B. Willberg, Nneka Nwokolo, Julie Fox, Sarah Fidler, W. Nicholas Haining, John Frater

**Affiliations:** ^1^Peter Medawar Building for Pathogen Research, Nuffield Department of Medicine, University of Oxford, Oxford, United Kingdom; ^2^Department of Infectious Diseases, Monash University, Melbourne, VIC, Australia; ^3^Department of Immunology, Harvard Medical School, Boston, MA, United States; ^4^Department of Pediatric Oncology, Dana-Farber Cancer Institute, Boston, MA, United States; ^5^Department of Paediatrics, University of Oxford, Oxford, United Kingdom; ^6^Division of Medicine, Wright Fleming Institute, Imperial College, London, United Kingdom; ^7^Oxford National Institute of Health Research Biomedical Research Centre, Oxford, United Kingdom; ^8^Chelsea and Westminster Hospital, London, United Kingdom; ^9^Department of Genitourinary Medicine and Infectious Disease, Guys and St Thomas’ National Health Service (NHS) Trust, London, United Kingdom; ^10^King’s College National Institute of Health Research (NIHR) Biomedical Research Centre, London, United Kingdom; ^11^Imperial College NIHR Biomedical Research Centre, London, United Kingdom; ^12^Discovery Oncology and Immunology, Merck Research Laboratories, Boston, MA, United States

**Keywords:** HIV, T cells, Primary HIV infection (PHI), Antiretroviral therapy (ART), Immune exhaustion

## Abstract

T cell dysfunction occurs early following HIV infection, impacting the emergence of non-AIDS morbidities and limiting curative efforts. ART initiated during primary HIV infection (PHI) can reverse this dysfunction, but the extent of recovery is unknown. We studied 66 HIV-infected individuals treated from early PHI with up to three years of ART. Compared with HIV-uninfected controls, CD4 and CD8 T cells from early HIV infection were characterised by T cell activation and increased expression of the immune checkpoint receptors (ICRs) PD1, Tim-3 and TIGIT. Three years of ART lead to partial – but not complete – normalisation of ICR expression, the dynamics of which varied for individual ICRs. For HIV-specific cells, epigenetic profiling of tetramer-sorted CD8 T cells revealed that epigenetic features of exhaustion typically seen in chronic HIV infection were already present early in PHI, and that ART initiation during PHI resulted in only a partial shift of the epigenome to one with more favourable memory characteristics. These findings suggest that although ART initiation during PHI results in significant immune reconstitution, there may be only partial resolution of HIV-related phenotypic and epigenetic changes.

## Introduction

The natural course of untreated HIV infection is characterised by the development of chronic immune activation and T cell dysfunction. These limit the prospect of immune-mediated viral control, and in particular the inability to clear latently-infected CD4 T cells that comprise the HIV reservoir. In an era where all those with HIV should be on antiretroviral therapy for life, this immune dysfunction is a significant hurdle for strategies towards a cure for HIV.

Following therapeutic success in the management of different cancers, T cell augmentation strategies, including immune checkpoint blockade, are being investigated as part of future treatment strategies for HIV (1, 2). In HIV infection, the expression of immune checkpoint receptors (ICRs) is pathologically high, and the association with altered T cell effector function (3–7), suggests similar potential for T cell reinvigoration.

In the development of HIV cure strategies, individuals who commence therapy early during primary HIV infection (PHI), are of particular interest because a smaller, less genetically diverse HIV reservoir (8–12) may make targeting this pool of latently-infected cells more achievable. Furthermore, immune function may be relatively preserved when ART is initiated in PHI, compared with chronic HIV infection (greater than 6 months after infection) (10, 13–18). However, despite early ART initiation, the dynamics of soluble and cellular measures of immune activation are unclear (9, 10, 19–21), with potential for persistent damage from the initial immunological insult.

During PHI, the expression of ICRs has been linked to subsequent CD4 T cell decline (5) and time to viral rebound following treatment interruption (22) – highlighting the clinical relevance of these markers during the early stages of infection. Interestingly, there is evidence that the expression of ICRs may differ between primary and chronic infection. PD-1 and TIGIT expression, as well as ICR co-expression, have been reported to increase as infection progresses from primary to chronic stages (7, 23–25). In one study, the proliferative defects associated with PD-1 expression were only observed in chronic infection, but not PHI (26), suggesting a temporally distinct role for this marker. Recent work in cohorts of acutely infected individuals suggests that ART initiation before viral peak (hyperacute infection) results in CD8 T cells with greater memory potential (27, 28), but also confirms that features of dysfunction are already present at this time (27).

Understanding the developmental timeline of T cell dysfunction during HIV infection is important for designing T cell augmentation strategies but remains largely unknown. It is not known if functional defects during PHI can be reversed with early ART, although observations from the transition from primary to chronic infection (3, 16, 29, 30) suggest that this may be possible. Furthermore, it remains unclear whether antigen-specific T cells present during ART have the potential to produce effective anti-viral responses *in vivo* if re-exposed to antigen.

We turned to the HIV Reservoir Targeting with Early Antiretroviral Therapy (HEATHER) cohort to characterise relevant immune parameters during primary HIV infection. This is a unique prospective UK cohort of treated primary HIV infection, within which we examined the effect of ART during PHI on the immune response. As well as examining memory differentiation, T cell activation, ICR and transcription factor expression patterns, we were able for the first time to explore with higher resolution the impact of ART in PHI on HIV-specific tetramer-sorted CD8 T cells using an assay for transposase-accessible chromatin (ATAC-seq), to determine the extent of change in chromatin accessibility following ART initiation, under-pinning the potential for full immune reconstitution.

## Results

### Study Participants

66 individuals who commenced treatment during PHI, as part of the HEATHER cohort, were included in this study. In brief, all were male with a median age of 34 (interquartile range; IQR 28 - 41) years at the time of ART start. They commenced ART a median of 29 (IQR 14 - 45) days following a confirmed HIV diagnosis, equating to a median of 52 (IQR 34 - 98) days following estimated seroconversion. Individuals in this cohort had a high median baseline VL (5.4 log_10_ copies/mL; IQR 4.4 - 6.4) and baseline CD4 count of 530 (IQR 406 – 652) cells/μL. Further clinical and demographic details of individuals included in this study are listed in **Table S1**, and described in detail elsewhere (31). For comparison, 10 healthy controls are included in this work (all were male; median age 35 [IQR 31 - 43] years) (**Table S1**).

### Expansion of Differentiated CD4 and CD8 T Cells During Primary HIV Infection Prior to ART

**Figure 1:**
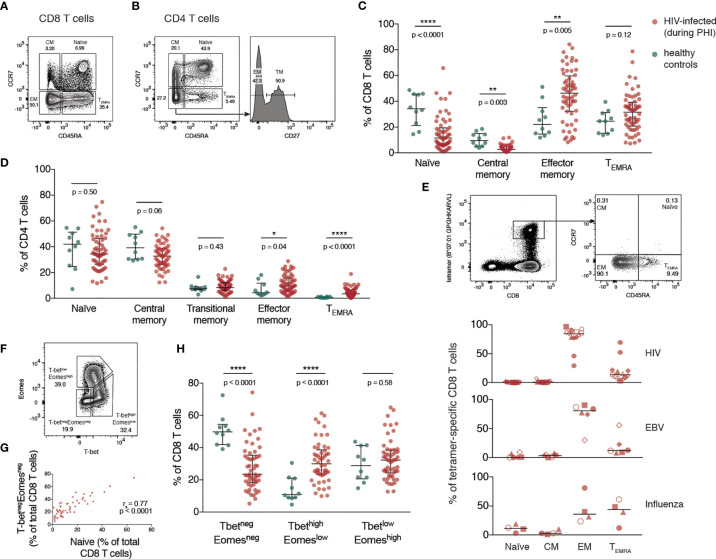
Expansion of differentiated CD4 and CD8 T cells during primary HIV infection. **(A)** Representative gating of CD8 T cell memory subsets. **(B)** Representative gating of CD4 T cell memory subsets. The percentage of T cells comprising each memory subset is shown in **(C)** (CD8 T cells) and **(D)** (CD4 T cells) for healthy individuals (n = 10; green) and HIV-infected individuals during PHI prior to ART (n = 60; red). Groups were compared using a Mann-Whitney test. **(E)** Memory phenotype of HIV, EBV and influenza-specific CD8 T cells from individuals (n = 9) during PHI. The panels on the top show representative HIV tetramer staining. Symbol shapes indicate tetramers from the same individual. EBV and influenza tetramers were not available for all individuals; for some individuals multiple different HIV tetramers were used and these are shown as separate data points. **(F)** Representative gating from one donor during PHI shows the division of CD8 T cells into 3 populations: T-bet^neg^Eomes^neg^, T-bet^high^Eomes^low^ and T-bet^low^Eomes^high^. **(G)** The frequency of T-bet^neg^Eomes^neg^ and naïve CD8 T cells are plotted (n = 51); data were tested using a Spearman correlation. **(H)** The frequency of each of T-bet^neg^Eomes^neg^, T-bet^high^Eomes^low^ and T-bet^low^Eomes^high^ populations in healthy controls (green; n = 10) and during PHI (red; n = 54) is shown. Groups were compared using a Mann-Whitney test. For all panels, bars indicate median and interquartile range. * indicates p < 0.05; ** indicates p < 0.01; **** indicates p < 0.0001.

The memory phenotype of CD4 and CD8 T cells was measured by flow cytometry (with gating as shown in **Figures 1A, B** and **Figure S1A**) during PHI (before ART was initiated) and in healthy controls. For both CD8 (**Figure 1C**) and CD4 T cells (**Figure 1D**) there was a relative increase in more differentiated memory subsets during PHI (shown in red) compared with healthy controls (in green). This was also assessed on HIV, EBV and influenza multimer-specific CD8 T cells for 9 individuals during PHI (additional gating shown in **Figure S1B**). **Figure 1E** shows that almost all HIV-specific T cells have an effector memory (EM) phenotype, reflective of changes in the bulk CD8 T cell pool.

The T-box transcription factors, T-bet and eomesodermin (Eomes), operate in concert in the development of effector T cell functions and have been implicated in the fate of exhausted CD8 T cells (32, 33). CD8 T cells were characterised with respect to their expression of T-bet and Eomes (gated as shown in **Figure 1F**). During PHI, the proportion of CD8 T cells expressing neither transcription factor (T-bet^neg^Eomes^neg^) correlated strongly with the proportion of naïve CD8 T cells gated on the basis of CD45RA and CCR7 (p < 0.0001, r = 0.77, **Figure 1G**). Naïve T cells do not express T-bet or Eomes (34), and it is likely that the T-bet^neg^Eomes^neg^ population, and the CCR7+/CD45RA+ populations are the same. Two subsets of antigen experienced CD8 T cells have been described with reciprocal expression of these markers: the more functional T-bet^high^Eomes^low^ cells, and T-bet^low^Eomes^high^ cells that show features of more terminal exhaustion (poor responsiveness to PD-1 blockade, poor polyfunctionality and high ICR co-expression) (32, 35). T-bet^high^Eomes^low^ cells were expanded during PHI compared with healthy controls (p < 0.0001, **Figure 1H**). The terminally exhausted T-bet^low^Eomes^high^ population showed the highest level of PD-1 expression (**Figure S2**).

### T Cell Activation and Increased Immune Checkpoint Receptor Expression During Primary HIV Infection Correlate With Disease Progression

**Figure 2:**
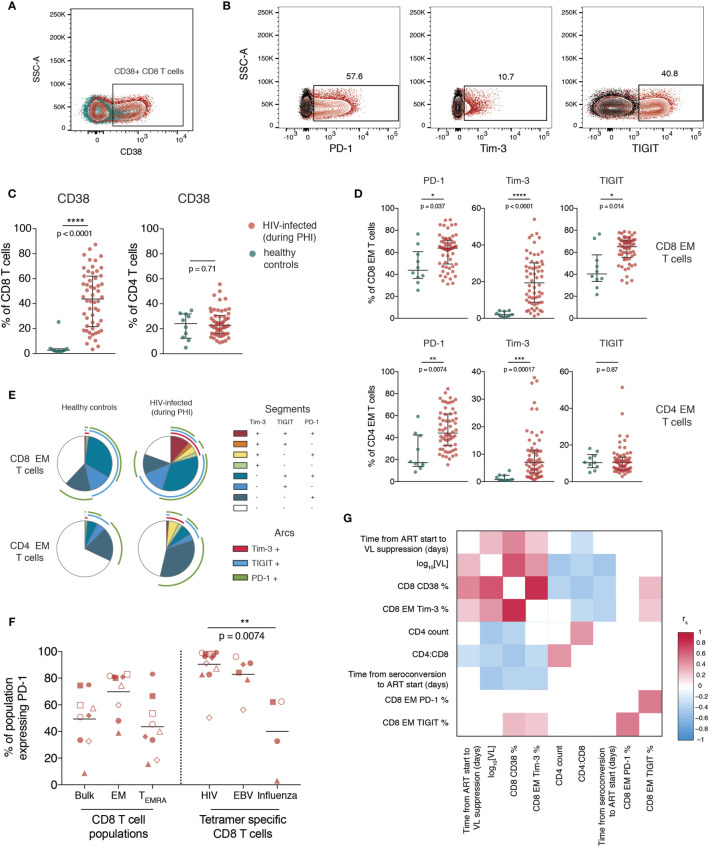
T cell activation and increased immune checkpoint receptor expression during primary HIV infection relates to measures of disease progression. **(A)** Representative staining of CD38 on CD8 T cells; plot shows one individual during PHI (red) overlaid over healthy control (green). **(B)** Representative gating of PD-1, Tim-3 and TIGIT; plots are gated on CD8 EM cells and show staining from one individual during PHI (red) overlaid over gating control (black) **(C)** Expression of CD38 on CD8 and CD4 T cells (n = 54 PHI, red; n = 10 healthy, green). Groups were compared using a Mann-Whitney test. **(D)** The expression of PD-1, Tim-3 and TIGIT on EM CD8 and CD4 T cells is compared between PHI (n = 60, red) and healthy control (n = 10, green). Expression was compared on all memory subsets using Mann-Whitney tests (complete data shown in **Figure S5**) and adjustment of p-values for multiple comparisons was performed to control the false discovery rate. Asterisks and listed p-values correspond to the adjusted p-value. **(E)** Co-expression of PD-1, Tim-3 and TIGIT on CD8 and CD4 EM cells from healthy controls and during PHI. Each segment represents the proportion of cells (median) with a different co-expression combination; arcs indicate which markers are in that combination. **(F)** PD-1 expression on bulk CD8 T cell populations, as well as tetramer specific CD8 T cells. Tetramer specific CD8 T cells were compared using a Kruskal-Wallis test (overall p = 0.0088) with subsequent pairwise comparisons using Dunn’s test. Symbol shapes indicate tetramers from the same individual. EBV and influenza tetramers were not available for all individuals; for some individuals multiple different HIV tetramers were used and these are shown as separate data points. **(G)** Correlations between CD8 EM expression of PD-1, Tim-3 and TIGIT during primary HIV infection and clinical measures of disease progression. The colour of each block corresponds to the correlation coefficient between any two variables. Variables have been ordered using hierarchical clustering. Correlation coefficients were calculated using the Spearman method with pairwise complete observations; only correlations significant at a p = 0.05 level are shown (other boxes are left blank). For all panels, bars indicate median and interquartile range. * indicates p < 0.05; ** indicates p < 0.01; *** indicates p < 0.001, **** indicates p < 0.0001.

The proportion of bulk CD4 and CD8 T cells expressing CD38 (**Figure 2A**), and the ICRs PD-1, Tim-3 and TIGIT (gated as in **Figure S1A** and **Figure 2B**) was then quantified. Our data shows that during primary infection, the proportion of CD8, but not CD4, T cells expressing CD38 was markedly elevated (**Figure 2C**).

Expression of ICRs is known to differ substantially between memory subsets (confirmed here in **Figure S3**) so the expression of these markers was explored on individual memory subsets rather than on bulk cells. During primary HIV infection, the expression of PD-1, Tim-3 and TIGIT was increased on EM CD8 and PD-1 and Tim-3 on EM CD4 T cells relative to healthy controls (**Figure 2D**; summary data showing this comparison for all subsets is shown in **Figure S4**). **Figure 2E** shows the co-expression of these three markers on the same subset and shows that this is due to both an increase in the proportion of cells expressing any ICR, and also the co-expression of these markers. In **Figure 2F** we confirm that at this early stage of infection, almost all HIV-specific CD8 T cells express PD-1.

The expression of CD38 and Tim-3 during PHI are closely related to markers of disease progression. This is shown in **Figure 2G**, which demonstrates positive relationships of CD38 and Tim-3 expression with baseline VL and an inverse relationship with CD4 count and CD4:CD8 ratio. PD-1 and TIGIT expression, while closely related to one another, do not exhibit this same, strong relationship with these clinical parameters.

### Incomplete Restoration of T Cell Phenotype Following ART Initiation During Primary HIV Infection

**Figure 3:**
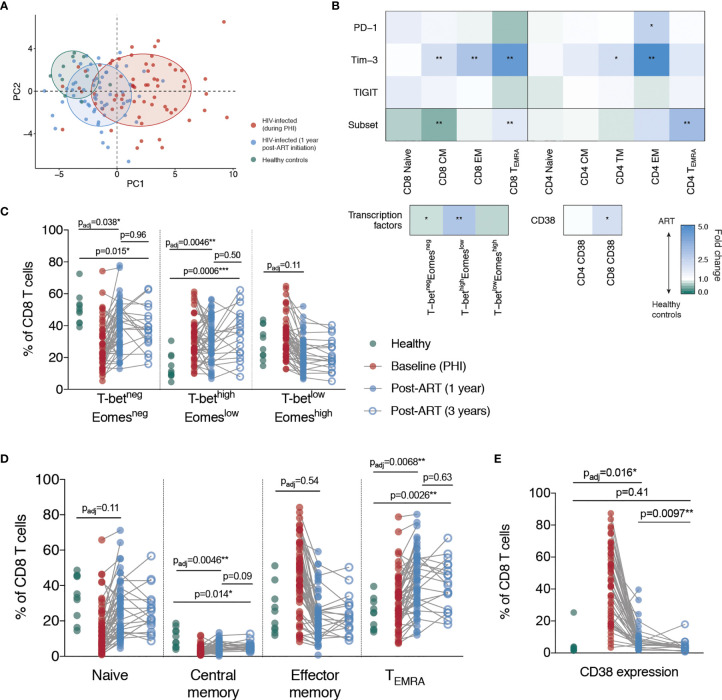
Partial but incomplete restoration of T cell phenotype following ART initiation during primary HIV infection. **(A)** Principle component analysis of all immune parameters measured here; plotted are the first two principle components (PC1 against PC2). For the construction of this PCA, missing immunologic measures were imputed using MissForest (36). Each point represents an individual from one of the following groups: healthy controls (green), primary HIV infection (red) and 1-year following ART initiation (blue). Ellipses have been added to aid in visual comparison and encompass 60% of values for each group. **(B)** Summary of comparisons between healthy controls and individuals following 1 year of ART. Blocks, each corresponding to an immune parameter, are shaded according to the fold difference between ART-treated individuals relative to healthy controls. The subset refers to the frequency of that memory subset as a proportion of bulk T cell pool, and the PD-1, Tim-3 and TIGIT rows refer to the percentage expression of those markers on that memory subset. Groups were compared using Mann-Whitney tests and adjustment of p-values for multiple comparisons was performed across all comparisons presented here to control the false discovery rate; asterisks correspond to the adjusted p-value. **(C)** T-bet and Eomes expressing CD8 T cells subsets. **(D)** CD8 T cell memory subsets. **(E)** CD38 expression on CD8 T cells. For **(C–E)** data are shown for healthy controls (n = 10, green), during PHI (C and E n = 54, D n = 60, red), following 1 year of ART (C and E n =6 0, D n = 58, blue closed circles) and 3 years of ART (n = 18, blue open circles). Comparisons between healthy controls and 1 year values were performed as in B (asterisks and listed p-values correspond to the adjusted p-value); additional tests involving 3 year timepoint are only shown for groups for which p < 0.05 in **(B)**. Other groups were compared using Mann-Whitney tests (unadjusted); the PHI group is shown as a visual aid and was not included in formal comparisons. For comparisons * indicates p < 0.05; ** indicates p < 0.01; *** indicates p < 0.001.

Principle component analysis was performed using all CD4 and CD8 T cell phenotypic features described thus far (**Figure 3A**); a full list of included variables is in **Table S2**. As expected, individuals during primary HIV infection and healthy controls cluster separately. The top five contributors to PC1 (which is largely driving this separation) are: the proportion of CD8 T cells expressing CD38, the proportion of Tim-3 expressing EM CD8 T cells, the proportion of Tim-3 (and then PD-1) expressing TM CD4 T cells and the proportion of Tim-3 expressing CD8 T_EMRA_ cells. When individuals who are treated from PHI with ART for one year are included in this analysis (blue), they cluster part-way between these other two groups – demonstrating partial but incomplete resolution of T cell phenotype compared with healthy controls.

To examine what markers were driving these observed differences between healthy controls and HIV-infected individuals following one year of ART, we performed a comparative analysis of all measured parameters. Our results demonstrate unresolved changes in T cell memory distribution and expression of CD38, Tim-3 and PD-1 (**Figure 3B**). We found that after one year after ART initiation, the proportion of CD8 T cells with a central memory (CM) and T-bet^neg^Eomes^neg^ phenotype remained significantly decreased relative to healthy controls (**Figures 3C, D**). The proportion of T_EMRA_ and T-bet^high^Eomes^low^ CD8 T cells was also elevated after one year. We therefore measured the same parameters after three years of ART in a subset of individuals (n=18; selected based on sample availability) to determine whether a longer duration of therapy would be more effective, however there was still a lack of resolution at this time-point (**Figures 3C, D**).

**Figure 4:**
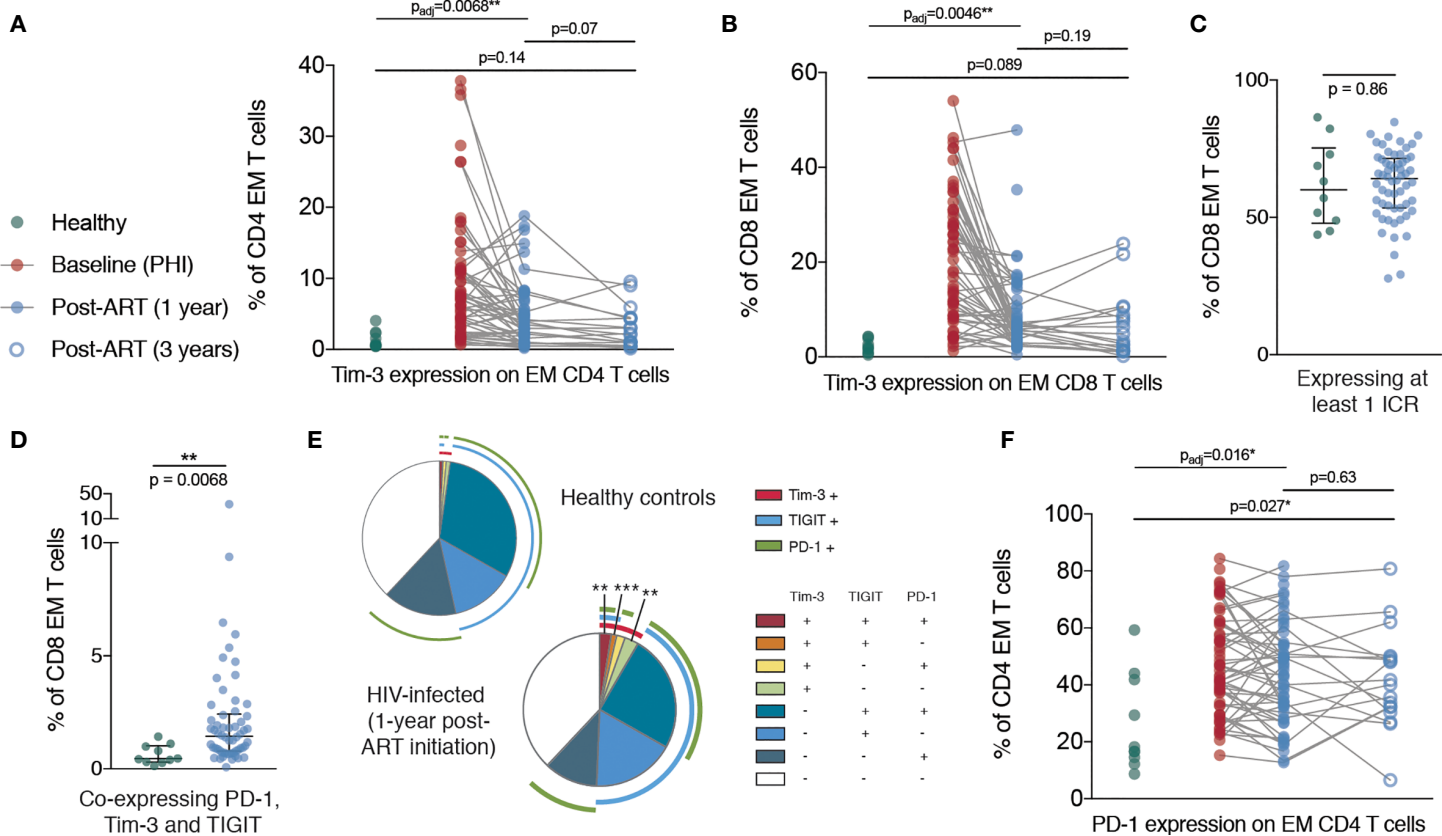
Persistent elevation of Tim-3 and PD-1 on memory T cells despite ART initiation during PHI. **(A)** Tim-3 expression on EM CD4 T cells are shown for healthy controls (n = 10, green), during PHI (n = 60, red), following 1 year of ART (n = 58, blue closed circles) and 3 years of ART (n = 18, blue open circles). **(B)** Tim-3 expression on EM CD8 T cells. The percentage of CD8 EM cells which express at least 1 ICR (PD-1 or Tim-3 or TIGIT) **(C)** or all three ICRs **(D)** was compared between healthy controls (green) and 1 year ART-treated individuals (blue). **(E)** Co-expression of ICRs on CD8 EM cells in healthy controls following 1 year of ART. Each segment represents the proportion of cells (median) with a different co-expression combination; arcs indicate which markers are in that combination. Co-expressing combinations (on all CD4 and CD8 memory subsets) were compared between healthy controls and ART treated individuals using a Mann-Whitney test; p-values were adjusted to control the false-discovery rate. Segments marked with an asterisk are significantly different between healthy controls and ART-treated individuals. **(F)** PD-1 expression on CD4 EM T cells. For **(A, B, F)** Comparisons between healthy controls and 1 year values were performed as in **Figure 3B** (asterisks and listed p-values correspond to the adjusted p-value); additional tests involving 3 year timepoint are only shown for groups for which p < 0.05 as in **Figure 3B**. Other groups were compared using Mann-Whitney tests (unadjusted); the PHI group is shown as a visual aid and was not included in formal comparisons. Asterisks and listed p-values correspond to the adjusted p-value. * indicates p < 0.05; ** indicates p < 0.01; *** indicates p < 0.001.

Despite a dramatic reduction in CD38 expression in the first year following ART initiation, CD38 expression on CD8 T cells was still elevated relative to healthy controls at this time. However, after three years of ART treatment, there was no significant difference in the expression of CD38 on CD8 T cells compared with HIV-negative controls, suggesting that this component of immune dysfunction was reversible (**Figure 3E**). Tim-3 expression was elevated at one year post-ART initiation on CD4 and CD8 EM T cells and had further decreased at three years to a level no longer significantly different to healthy controls (**Figures 4A, B**). This suggests that in contrast to the more fixed T cell phenotype skewing and changes in T-bet and Eomes expression, there is heterogeneity in those phenotypic characteristics which show reversibility on ART.

In addition, after one year of ART, whilst the frequency of CD8 EM cells expressing at least one ICR did not differ from healthy controls, we observed an increase in Tim-3 expression which was associated with an increase in overall ICR co-expression (**Figures 4C–E**). Strikingly, the proportion of CD4 EM T cells expressing PD-1 was increased in primary infection and remained almost unchanged even three years after ART initiation (**Figure 4F**).

### HIV-Specific CD8 T Cells Exhibit Exhaustion-Associated Epigenetic Changes During PHI Which Alter Following Initiation of ART

**Figure 5:**
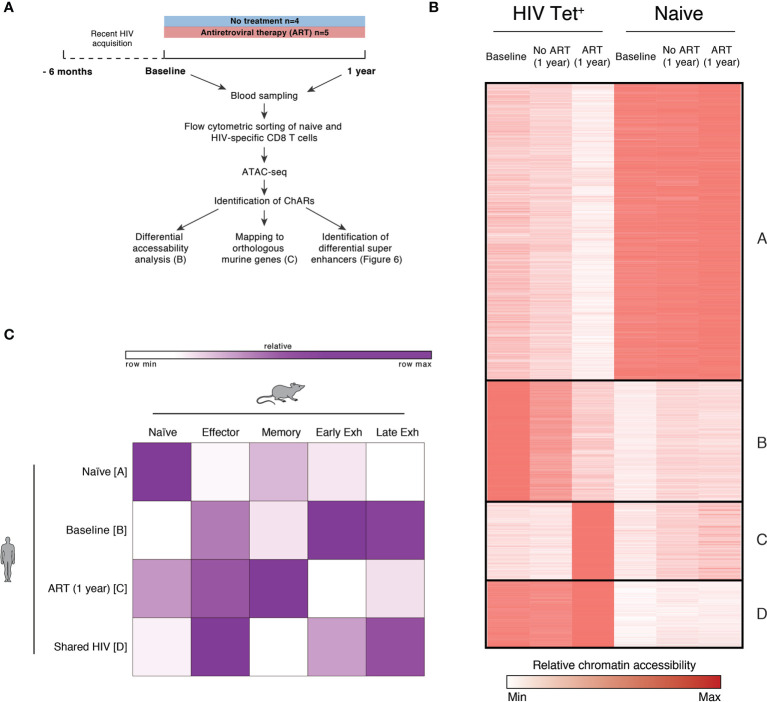
HIV-specific CD8 T cells exhibit exhaustion-associated epigenetic changes during PHI but not following 1 year of ART. **(A)** Schematic outlining the experimental setup used for the epigenetic profiling of naïve and HIV-specific CD8+ T cells. **(B)** k-means clustered heatmap of chromatin accessibility within modules of chromatin accessible regions (ChARs) (rows) showing 81,138 regions (out of a total universe of 172,174) that were differential at an FDR threshold < 0.05 between at least one of all possible pairwise comparisons. The quantitative differential analysis was run using all samples that passed QC and individual patients as biological replicates; for the construction of this heatmap values for each condition was averaged across the patients. Values have been row normalized to account for the fact that different open chromatin regions can have different baseline accessibilities. **(C)** Fold enrichment of regions orthologous to mouse naïve, effector, memory and exhaustion enhancers in human in clusters identified in B, which are most prominent in naïve (cluster A), or HIV-specific CD8 T cells at baseline (cluster B), following one year of ART (cluster C) or in both conditions (cluster D).

In view of the heterogeneity observed after three years of ART, we next set out to determine if exhaustion-associated changes in chromatin accessibility within HIV-specific T cells could be reversed when ART is initiated during PHI. We sorted HIV-specific CD8 T cells (**Figure S2B**) and performed ATAC-seq, a high throughput method for sequencing chromatin accessibility (37, 38). To determine if exhaustion-associated epigenetic changes are already present during PHI, we sorted cells from individuals during PHI as well as matched individuals after one year of infection, both untreated and following 12 months of ART (**Figure 5A** and **Figure S5**).

Assays were performed from five individuals from the HEATHER cohort who initiated ART during PHI, and four individuals from a previously completed study [SPARTAC (39)] who were followed from primary to chronic infection without ART. Individuals in both groups were similar with regards to key demographic variables (including age, country of recruitment, viral subtype and baseline VL, CD4 count). These demographic and baseline clinical characteristics of these individuals are summarised in **Table S3** and **Figure S5**. The HLA type and epitope sequences of tetramers used for each individual are shown in **Table S4**. Individuals were identified for the inclusion in this analysis on the basis of sample availability, and the presence of identifiable HIV-specific T cell responses. Plasma viruses (at untreated timepoints) were sequenced *via* Sanger sequencing to confirm lack of immune escape and ongoing antigen exposure, and are shown in **Table S4**; two tetramer responses were excluded because of identified escape mutations at baseline which might have abrogated tetramer binding.

Chromatin accessible regions (ChARs) identified *via* ATAC-seq were compared between naïve and HIV-specific CD8 T cells at baseline, following one year of ART, and one year without treatment. **Figure 5B** shows a k-means clustered heatmap of regions that were differential at an FDR threshold < 0.05 between at least one of all possible pairwise comparisons. As expected, we observe distinct partitioning of naïve and non-naïve (HIV-specific) T cells. HIV-specific CD8 T cells from individuals at baseline (during PHI) and chronic infection (1 year without treatment) share similar chromatin accessibility profiles, suggesting that epigenetic features of chronic HIV infection are established during the very early stages of infection. While T cells from individuals following 1 year of ART show some shared chromatin accessible regions with both baseline and untreated HIV subjects there are, however, a substantial number of regions in clusters B and C with decreased or increased accessibility compared with baseline indicating epigenetic remodelling after commencement of ART. To explore potential functional implications of this remodelling, two further analyses were performed directly comparing individuals in primary infection with those post-ART.

A signature of epigenetic features associated with exhaustion in LCMV models has been previously identified using ATAC-seq (40). Open chromatin regions from HIV-specific T cells from individuals with chronic HIV infection were mapped to orthologous counterparts identified in murine models, highlighting that these cells had an epigenome resembling exhausted CD8 T cells (40). **Figure 5C** quantitates the degree to which the clusters identified in **Figure 5B** can be mapped to orthologous regions identified in murine naïve, effector, memory and exhausted CD8 T cells. As expected, human naïve CD8 T cells showed the most similarity in chromatin accessibility to those regions identified in mouse naïve cells (compared with those identified in memory or exhausted T cells). HIV specific T cells during primary HIV infection (clusters B and D) showed the greatest chromatin accessibility in exhaustion-specific regions (particularly late exhausted regions). Whereas we see distinct differences in cluster B between baseline or untreated individuals versus one year of ART, there is much less evidence for any resolution on ART within cluster D (**Figure 5B**). This contrasts with those HIV-specific cells still present following 1 year of ART (cluster C) which showed more epigenetic similarity to murine memory T cells.

**Figure 6:**
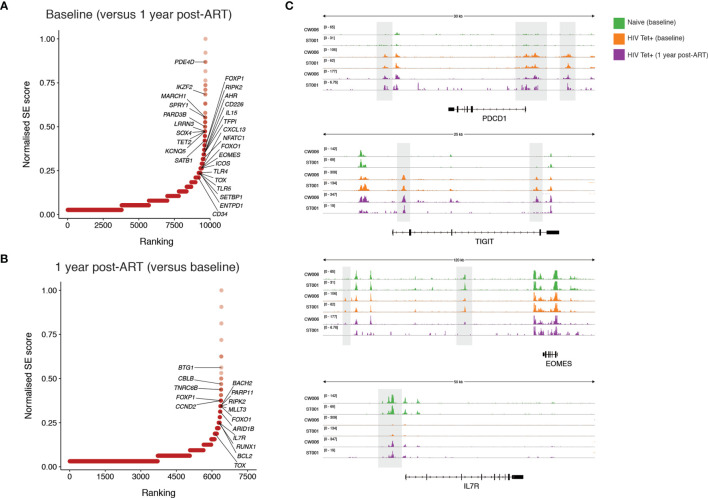
Identification of super-enhancers which are differentially accessible prior to and following one year of ART initiated during PHI. Super-enhancer elbow plots based on chromatin accessible regions (ChARs) within HIV-specific CD8 T cells which are differentially accessible between baseline and following one year of ART including those that show increased accessibility at baseline compared with one year following ART **(A)** and which show increased accessibility following one year of ART compared with baseline **(B)**. **(C)** Representative ATAC-seq tracks at the *PDCD1, TIGIT, EOMES and IL7R* gene loci. Numbers in brackets represent the number of reads for peaks that are being visualised.

To further probe functional implications of the epigenetic differences identified pre- and post-ART we next focused on the genes associated with differentially accessible regions. Super-enhancers are transcriptional regulatory complexes comprised of clusters of enhancers, which are usually responsible for the transcription of genes involved in determination of cell identity (41, 42). Identification of super-enhancers based on chromatin accessibility was performed. The accessibility of predicted super-enhancers was defined and compared between individuals pre-and post-ART. Genes with predicted differential super-enhancers accessible at baseline (**Figure 6A**) and following 1 year of ART (**Figure 6B**) were identified. Amongst highly ranked genes with increased super-enhancer accessibility during PHI were genes associated with T cell activation (*PDE4D*, *CD226, IL15*), regulation of lymphocyte development (*SATB1*, *SPRY1*) and several genes that have been linked to HIV control or anti-HIV cytotoxicity [*PARD3B* (43), *KCNQ5* (44), *IKZF2* (45)]. Compared with baseline, super-enhancers with increased accessibility at one year post-ART were identified in genes with key roles in cell survival and genes with key roles in memory formation (*BCL2*, *IL7R* [example data shown in **Figure 6C**]), as well as negative regulators of T cell activation/proliferation (*BTG1*, *CBLB*). There was substantial change in the chromatin accessibility around the locus for *TOX*, a transcription factor thought to play a critical role in T cell exhaustion (46), with this area showing increased accessibility at both timepoints. Additionally, the super-enhancer associated with a well-characterised preferential HIV integration site [*BACH2* (47, 48)] was identified to have increased accessibility at this timepoint. Despite phenotypic decreases in PD-1 and TIGIT expression at 1 one-year post-ART on bulk CD8 T cells, epigenetic remodelling pre- and post-ART around these genes in HIV-specific T cells was not observed (**Figure 6C**). This is in contrast to *EOMES* where decreased chromatin accessibility following 1 year of ART (evident in **Figure 6A** with example data shown in **Figure 6C**) relative to baseline mirrors the shifts in protein expression observed in bulk CD8 T cells.

## Discussion

We have characterised the phenotypic and epigenetic features of T cells during PHI and how these are impacted by the initiation of ART early in HIV infection. Primary HIV infection was characterised by major shifts in the differentiation of bulk CD4 and CD8 T cells, in particular by the expansion of activated CD8 T cells with an EM phenotype, which has been well-described in several previous studies (26–28, 30). Our study also confirmed previously described increases in the expression of the ICRs PD-1, Tim-3 and TIGIT (4, 5, 7, 25, 49–52) on memory T cells.

Several phenotypic features of T cell exhaustion measured here (including the expression of PD-1 on memory T cells) have been shown to progress in the transition from primary to chronic HIV infection in the absence of treatment (25, 26, 30). Here, we have observed that the initiation of ART only partially restores these features to a phenotype similar to that seen in healthy controls.

Overall, we observed a partial but incomplete resolution of these phenotypic characteristics of untreated HIV infection with early ART. Following one year of ART, we found relative expansion of the T_EMRA_ T cells which persisted even after three years of treatment, suggesting a permanent alteration in the T cell pool or low-level persistent antigenic stimulation driving the differentiation of these cells. Indeed, there is now evidence of ongoing T cell recognition of HIV antigens during suppressive ART (53), further supporting that this may be a driver of immune activation and inflated CD8 T cell numbers in many individuals on ART. In contrast, the initiation of early ART resulted in near-normalisation of ICRs on bulk memory T cells after one (in the case of PD-1 and TIGIT) and three years (Tim-3) of treatment. A similar observation was made with CD8 T cell activation measured by CD38 expression. A notable exception to this was the expression of PD-1 on EM CD4 T cells which remained unchanged despite three years of ART, consistent with earlier findings (25, 26).

Earlier initiation of ART has been linked with more favourable clinical outcomes (54), but whether it can resolve HIV-associated chronic inflammation remains unclear (9, 10, 19–21). As a major driver of HIV-related morbidity, even during successful ART (55), there is significant interest in whether early initiation of therapy can normalise chronic immune activation and T cell dysfunction. Here, we have observed near normalisation of phenotypic markers of T cell activation (CD38 and ICR expression) after 3 years with ART initiation during PHI, and further longer term data will be invaluable in understanding the full potential for resolution of T cell dysfunction with early ART.

When interpreting these findings, it is important to consider that changes observed in bulk memory populations may not be entirely reflective of the HIV-specific pool; the degree to which bystander activation, and ICR expression, occurs has been previously studied. Certainly it has already been shown that during untreated HIV infection, upregulation of PD-1, TIGIT and Tim-3 occurs on HIV-specific T cells, as well as being observed in bulk populations (3, 4, 6, 7, 50, 56) (as was shown here). HIV-specific T cells are felt to contribute substantially to this; as evidence of this PD-1, Tim-3 and TIGIT have been shown to be expressed at higher levels on HIV-specific CD8 cells compared with other viral-specific cells from the same individual (3, 4, 6, 50, 57).

It is possible that a reduction in the size of the HIV-specific pool in the absence of ongoing antigen means that any sustained ICR expression is no longer evident in the bulk memory pool, but might have been able to be measured on HIV-specific T cells. Alternatively, a reduction of antigen to drive ongoing expression may also have decreased expression on HIV-specific T cells. Whilst not directly measured here, this latter idea is supported by previous findings that PD-1 expression is decreased on HIV-specific T cells following viral escape from their cognate epitope (29, 58). These two possibilities impact on how sustained epigenetic changes following ART around the PD-1 and TIGIT loci (**Figure 6C**) can be understood. Regardless, our findings of sustained epigenetic changes within HIV-specific T cells suggest that epigenetic changes around these loci are ongoing despite ART, and have implications for the potential to use antigen experienced HIV-specific T cells therapeutically.

There is considerable interest in the development of immunotherapeutic approaches to augment the anti-HIV CD8 T cell response as part of an HIV cure. Critical for the success of these approaches is understanding if CD8 T cells primed during viral replication and before ART is commenced have the potential to regain functional characteristics. In SIV-infected macaques treated with long term ART, blockade of PD-1 and/or CTLA-4 does not improve proliferative or cytolytic SIV-specific CD8 T cell responses, despite high levels of PD-1 expression on these cells (59). Exhausted T cells in murine models have been shown to have transcriptomic, metabolic and epigenetic features that are distinct from effector T cells (40, 60, 61) - providing evidence that T cell exhaustion represents a specific functional state or cell fate. Indeed, PD-1 expressing T cells have shown limited epigenetic remodelling (62) or upregulation of alternative ICRs (63) in response to PD-1 blockade, suggestive of an underlying cellular state which is not readily modifiable. Further supporting this concept of epigenetic inflexibility, demethylation around the *PDCD1* locus (which encodes PD-1) has previously been observed during PHI and does not revert following virological suppression with ART initiated during chronic infection (64); findings that were confirmed here. We have demonstrated that epigenetic features of HIV-specific T cells during chronic infection (when dysfunction is well-characterised) are already evident during PHI, defined as the first 6 months following HIV infection. If stable, this would suggest that early ART initiation may not be sufficient to preserve a T cell pool with full functional potential.

With the initiation of ART during PHI, however, we also observed a shift in the epigenetic landscape of HIV-specific T cells towards one with more features of memory T cells including increased accessibility of transcriptional regulators of key genes associated with enhanced cell survival and functional memory responses (*BCL2*, *IL7R*). These changes could represent partial epigenetic remodelling. More likely, however, is that this represents the preferential persistence of cells with memory characteristics where, in the absence of antigen, the highly activated, short-lived expanded effectors that characterise HIV infection do not survive. This is supported by the decrease in T-bet^low^Eomes^high^ cells following ART initiation, a population which (in mice) cannot self-renew and requires ongoing antigenic exposure to be replenished from progenitor populations including T-bet^low^Eomes^dim^ cells (32).

We identified a high degree of change in chromatin accessibility around the *TOX* locus, which had increased differential accessibility at both time points. Super-enhancers consist of many smaller component enhancers, and this intriguing result likely reflects varying effects at these sites. Indeed there is evidence of that redundancy in enhancer sites is common (65); this may drive the phenotypic stability of T cell exhaustion making this harder to reverse through ART.

Further functional insights were gained by the mapping of human regions of chromatin accessibility to the orthologous genes from murine models. A recent study has taken genes identified using these same murine epigenetic data sets (40, 62) and transcriptomic studies to identify a candidate set of proteins most associated with exhaustion (66). Surface and intracellular proteins measured by mass-cytometry in individuals with chronic HIV infection showed patterns of expression that were associated with functional exhaustion (measured by chemokine and cytokine production), viral load and CD4:CD8 ratio (66). These data further support the relevance of epigenetic changes previously identified (40) (and utilised here) to T cell exhaustion and disease progression during HIV infection.

T cells during untreated chronic infection proliferate poorly, have low cytotoxicity, and low polyfunctionality (16, 29, 30, 67, 68). The timing of development of T cell dysfunction, and when interventions could be best employed to redirect T cell functionality, is an area of key interest. Recent studies of hyperacute HIV infection have revealed that HIV-specific CD8 T cells from individuals who receive ART before viral load peak (Fiebig I and II) have improved survival potential with increased expression of Bcl-2 and CD127 (IL-7R) (27, 28) compared with those who start at late stages of infection. CD127 expression during PHI has been shown to inversely correlate with viral load set point (3), directly invoking a role for this pathway in T cell mediated viral control. Here, we demonstrate an increase in chromatin accessibility around super-enhancers for these two key pro-survival genes (*BCL2* and *IL7R*) with initiation of ART during PHI. Individuals used for tetramer sorting experiments had a median of 32 (range 16 – 165) days between estimated seroconversion and sampling, and thus viral load has likely peaked. Overall, epigenetic analyses suggest that ART initiation during PHI is associated with the development of HIV-specific T cell memory pool with favourable memory characteristics.

This work has been performed within a large, well-characterised and contemporary cohort of PHI allowing clear characterisation of T cell phenotypes. There are some limitations to this work. Firstly, as a cohort study there is some variation, albeit small, in the time intervals between the baseline study visit and time of ART initiation. Secondly, while this work has made an effort to characterise a large number of aspects of T cell activation, memory differentiation and ICR expression, it largely focuses on bulk, rather than HIV-specific T cells and does not contain any measures of T cell function. In addition, epigenetic signatures consist of a number of features in addition to chromatin accessibility which are not profiled here. The epigenetic findings reported, however, demonstrate a unique approach to profiling CD8 T cell potential, and lay the groundwork for future experiments into functional potential of HIV-specific T cells following ART commenced during PHI.

Primary HIV infection is characterised by a large expansion of activated CD4 and CD8 T cells with an effector memory phenotype and with increased ICR expression. Here we have shown that ART initiation at this time is associated with partial resolution of ICR expression, and persistent shifts in memory composition of these T cell pools consistent with prior antigen exposure. Exhaustion-associated epigenetic features in HIV-specific CD8 T cells seen in chronic HIV infection are already evident during PHI, and although ART initiation at this time is associated with a change in the epigenome towards one with more functional, memory characteristics, this recovery is not complete.

## Methods

### Participant Information

HEATHER is a prospective observational cohort study of individuals who commence ART (and remain on uninterrupted therapy) within 3 months of the date of HIV diagnosis during PHI. Individuals are considered to have PHI if they meet any of the following criteria: HIV-1 positive antibody test within 6 months of a HIV-1 negative antibody test, HIV-1 antibody negative with positive PCR (or positive p24 Ag or viral load detectable), RITA (recent incident assay test algorithm) assay result consistent with recent infection, equivocal HIV-1 antibody test supported by a repeat test within 2 weeks showing a rising optical density or having clinical manifestations of symptomatic HIV seroconversion illness supported by antigen positivity. The time of seroconversion was estimated as the midpoint between the most recent negative or equivocal test and the first positive test for those who met relevant criteria and as the date of test for all other participants. Individuals with co-existent hepatitis B or C infection are not eligible for inclusion in HEATHER. CD4 count, CD8 count and VL were measured as part of routine clinical care with baseline VL and CD4 and CD8 counts taken as the earliest value prior to the initiation of ART. Samples from the HEATHER trial are used for the majority of experiments presented here.

SPARTAC (EudraCT Number: 2004-000446-20) was a multi-centre randomised controlled trial of short course antiretroviral therapy during PHI, which completed follow up in 2010. The full inclusion criteria and details of the SPARTAC trial are published elsewhere (39). PHI was defined and estimated date of seroconversion calculated similarly to above. In this work, samples were used at the time of enrolment (baseline) or following 1 year from 4 individuals who did not receive ART during this period.

### HLA Typing

HLA typing was performed to intermediate resolution using PCR with sequence specific primers (PCR-SSP).

### Flow Cytometry

An estimated 1 x 10^7^ cryopreserved peripheral blood mononuclear cells (PBMCs) were thawed in RPMI-1640 medium supplemented with 10% FBS, L-glutamine, penicillin and streptomycin (R10) containing 2.7 Kunitz units/mL of DNAse (Qiagen). Cells were stained in BD Horizon Brilliant Stain Buffer (BD) containing the following antibody panels and Live/Dead Near IR at a 1 in 300 dilution (Life Technologies) at 4°C for 30 minutes. Staining volumes were scaled directly so that no more than 5 x 10^6^ cells were stained per 50μL volume.

Panel 1 - CD3 Brilliant Violet (BV) 570 (UCHT1), CCR7 Pacific Blue (G043H7), CD27 AlexaFluor700 (M-5271)[all BioLegend], CD4 BV605 (RPA-T4), CD8 BV650 (RPA-T8)[all BD], PD-1 PE-eFluor610 (eBioJ105), CD45RA FITC (HI100), TIGIT PerCP-eFluor710 (MBSA43)[all eBioscience] and Tim-3 PE (344823)[R&D].

Panel 2 - CD3 BV570, CD38 AlexaFluor700 (HB-7) [BioLegend], CD4 BV605, CD8 BV650, PD-1 PE-eFluor610 and Tim-3 PE. Following this, cells were washed twice prior to fixation and permeabilisation with Foxp3 Buffer Set (BD) as per manufacturer’s directions with reduced volumes to facilitate staining in 96-well plates. Staining for intracellular epitopes was performed at room temperature for 30 minutes in PBS containing 0.5% BSA and 0.1% sodium azide with the following antibodies: T-bet FITC (4B10)[BioLegend] and Eomes eFluor660 (WD1928)[eBioscience].

For both panels, cells were washed twice, fixed with 2% formaldehyde for 30 minutes, then washed twice and resuspended in phosphate buffered saline (PBS) for acquisition.

All samples were acquired on a LSR II (BD) the day after staining. The same machine was used for all experiments with daily calibration with Cytometer Setup & Tracking beads (BD) to maximise comparability between days. Rainbow Calibration Particles (BioLegend) were also used for cohort phenotyping to minimise batch-to-batch variability. Data were analysed using FlowJo Version 10.8.0r1 (Treestar).

### Viral Sequencing

Sanger sequencing of plasma viral *gag* and *nef* genes was performed in several fragments using nested PCRs, similar to previously described (69, 70). cDNA synthesis and amplification were performed together using a One-Step RT-PCR System (Invitrogen). 4µL of RNA template was added into final reaction volume of 50µL containing primers at 200nM (gagC5OP and gagC3OP or nef5OP and nef3OP; sequences as in **Table S5**), an enzyme mix containing Platinum *Taq* Polymerase and SuperScript III RT (Invitrogen) and 1X Reaction Buffer (supplied with enzymes). Reactions were cycled on a thermocycler as follows: 55°C for 30 minutes; 94°C for 15 seconds, followed by 40 cycles of: 94°C for 15 seconds, 55°C for 30 seconds and 68°C for 45 seconds. Samples were then held at 68°C for 5 minutes.

A second-round, nested amplification was performed using 4µL of amplified DNA template from above step in a final reaction volume of 50µL containing forward and reverse primers at 200nM (as listed in **Table S5**), 0.8mM dNTPs (0.2µM each of dATP, dCTP, dGTP and dTTP; ThermoScientific), 1.5mM MgCl_2_, 0.2U/µL *Taq* Polymerase (Invitrogen) and 1X PCR buffer (supplied with enzyme). Reactions were cycled on a thermocycler as follows: 94°C for 2 minutes; 40 cycles of 94°C for 15 seconds, 55°C for 30 seconds and 72°C for 90 seconds; held at 72°C for 5 minutes.

PCR products were purified to remove residual dNTPs and primers using the QIAquick PCR Purification Kit (Qiagen) as per manufacturer’s instructions. Forward and reverse sequencing reactions were set up for each sample containing 7µL of amplified PCR product, one of the primers used for second-amplification at 330nM and the BigDye Terminator v3.1 Cycle Sequencing kit (ThermoFisher) with 0.25X Ready Reaction Mix and 0.75X reaction buffer (total reaction volume of 10µL). Reactions were cycled on a thermocycler as follows: 96°C for 30 seconds; 30 cycles of 96°C for 30 seconds, 50°C for 15 seconds and 60°C for 4 minutes.

2µL of 3M sodium acetate, 50µL of 100% ethanol and 10µL of water was added to the reaction product; this was held at room temperature for 30 minutes to precipitate the reaction product out of solution. The sample was centrifuged and supernatant removed prior to washing twice with 200µL of 70% ethanol. The pellet was dried on a thermocycler at 95°C for 1 minute, prior to being read using an Applied Biosystems 3730xl DNA Analyzer. Trace files were checked and manually edited for mixed bases using Sequencher v5.1 before alignment with the HIVAlign tool (Los Alamos National Laboratory).

### Multimer Staining and Cell Sorting

Biotinylated monomers of MHC Class I/peptide complexes were custom produced for HIV peptides of interest by Anette Stryhn Buus (immunAware, Laboratory of Experimental Immunology, University of Copenhagen, Denmark), using previously published methods (71). Tetramerisation was performed by the addition of Streptavidin-APC (Invitrogen), ExtrAvidin-PE or ExtrAvidin-FITC (both Sigma) to biotinylated monomers at a 1:4 molar ratio. For influenza and EBV epitopes, pre-conjugated MHC Class I dextramers were used (Immudex).

Cryopreserved PBMCs were thawed in R10 with 10mM HEPES and 0.001% β-mercaptoethanol, after which CD8 T cells were enriched by negative magnetic selection using the MACS CD8+ T Cell Isolation Kit (Miltenyi). CD8 T cells were resuspended at a concentration of 1 x 10^8^ cells/mL in isolation buffer containing 10% Human TruStain FcX (Fc Receptor Blocking Solution; BioLegend) and multimers (HIV tetramers at 30nM and EBV/influenza dextramers at 3.2nM) for 20 minutes.

Cells were washed and resuspended in isolation buffer containing 0.5X BD Horizon Brilliant Stain Buffer (BD) containing LiveDead Near IR and the following antibodies: CCR7 PerCP-Cy5.5 (G043H7), CD3 BV510 (OKT-3), CD45RA BV785 (HI100), CD8a BV711 (RPA-T8), CD95 (Fas) BV605 (DX2) and PD-1 BV421 (EH12.2H7) [BioLegend]. Surface staining was performed for 30 minutes at 4°C after which cells were washed twice with isolation buffer and strained through a 30μm nylon mesh prior to cell sorting. Up to 80,000 cells were sorted using a FACSAria (BD) into 400μL PBS with 10% FBS in DNA LoBind 1.5mL microcentrifuge tubes (Eppendorf) and processed immediately for ATAC-seq.

### Assay for Transposase Accessible Chromatin Sequencing (ATAC-Seq)

ATAC-seq was performed as previously published (37, 38, 40). Cells were pelleted at 400*xg* at 4°C for 8 minutes and supernatant was carefully removed by pipetting. A combined cell lysis and transposition step was performed by resuspending the cell pellet in 50µL reaction mix comprising 1X TD buffer, 1:20 dilution of Tn5 transposase (both from the Nextera DNA Sample Preparation kit, Illumina) and 0.01% digitonin. Where fewer cells were included, the reaction volume was directly scaled (with a minimum volume of 5µL) to match cell input. This was incubated at 37°C for 30 minutes in a ThermoMixer (Eppendorf) with agitation at 300rpm.

Following transposition, DNA was purified using a MinElute Reaction Cleanup kit (Qiagen) with double elution performed at 50°C into water.

Amplification of adapter-ligated DNA fragments was then performed by PCR. 20µL of purified DNA template was added into final reaction volume of 50µL containing 1.25µM each of Nextera forward and reverse PCR primers to Tn5-ligated adapters (from Nextera XT Index Kit, Illumina) and 1X NEBNext High-Fidelity PCR Master Mix (New England Labs). Primers contain barcoding sequences to allow multiplexing of sequencing and primer combinations were chosen to ensure desired libraries could be sequenced together. Reactions were cycled on a thermocycler as follows: 72°C for 5 minutes; 98°C for 30 seconds; 13-18 cycles of 98°C for 10 seconds, 63°C for 30 seconds and 72°C for 1 minute; hold at 4°C. The minimum number of cycles required for adequate amplification was used; this was estimated based on input cell number.

PCR reaction clean-up was performed in the 96 well PCR plate using SPRI (solid phase reversible immobilisation) beads (Agencourt AMPure XP purification system; Beckman Coulter) at a 1:1 ratio of beads to sample.

Library quality was checked *via* TapeStation. If further amplification was required, additional PCR cycles and bead cleanup as above were performed. Quantification of libraries was performed by qPCR using the KAPA Library Quantification Kit for Illumina platforms (KAPA Biosystems) as per the manufacturer’s instructions. Libraries were pooled and multiplexed libraries sequenced with 2 x 37 nt paired-end sequencing on a NextSeq 500 (Illumina; 75 cycle high-output kit).

### Analysis of ATAC-Seq Data

Sequencing reads were demultiplexed into fastq files using bcl2fastq (v2.19.1.403). Quality trimming and primer removal within raw fastq files were done with Trimmomatic (v0.36) using the following parameters: LEADING:15 TRAILING:15 SLIDINGWINDOW:4:15 MINLEN:36. Sequencing quality was checked using FastQC (v0.11.3, Babraham Bioinformatics). Reads were aligned to hg19 using Bowtie 2 (v2.2.9), with only reads that mapped to a unique position in the genome retained, and a maximum included fragment length of 1000bp. Duplicate reads were marked using Picard (v2.8.0) and checked for contaminating sequences. Reads mapping to blacklisted areas of the genome [as identified in the ENCODE project (72) and the Buenrostro lab (37, 38)], as well as ChrM, were removed. Aligned reads were shifted +4bp or -5bp as appropriate. All samples were assessed for library quality according to ENCODE guidelines (https://www.encodeproject.org/atac-seq/) including the calculation of the fraction of reads in called peak regions (FRiP score) as well as TSS enrichment.

For the identification of peaks, Binary Alignment/Map (BAM) files of biological conditions were merged using SAMtools (v1.3). Peak calling was performed using MACS2 (v2.1.1), with a false discovery rate (FDR) of 0.001. The peak “universe” was then defined as the union of these peaks across biological conditions, with overlapping regions merged. Quantitation of the number of cut sites within each peak region was performed using BEDTools (v2.26.0).

DESeq2 (v1.26.0) was used to normalize the counts matrix and perform differential accessibility analysis between all relevant comparisons. For a given comparison, an FDR cutoff of 0.05 was used to determine differential ChARs.

Orthologous mouse ChARs (mm10) were mapped to the human genome (hg19) as described (40). Since the mapping algorithm requires input regions in mm10, the UCSC liftover tool was applied to ChARs to transfer them onto mm10 from mm9. All mouse peaks partitioned into 5 categories based on previously published clustering (40). Then, for each set of consensus peaks in the HIV dataset, the hypergeometric fold enrichment was calculated separately for the 5 categories of mouse orthologous peaks (naïve, effector, memory, early exhaustion and late exhaustion).

Detailed procedures for the identification and validation of predicted super-enhancers from ATAC-seq data are described elsewhere (41, 73). In brief, the number of differential open chromatin regions associated with each gene were calculated using GREAT (v3.0.0, http://bejerano.stanford.edu/great/public/html/) under default settings. The resultant gene list was ranked from highest to lowest based on number of regions, representing putative components of a super-enhancer, and then plotted against the total number of regions to generate a characteristic elbow plot. The x- and y-axes were then scaled from 0-1 and the inflection point in the curve was determined by using the findElbow tool within the ChemoSpecMarkeR package (Bryan A. Hanson). All genes with a higher number of regions than the inflection point were defined as SE-associated.

### Statistics

ATAC-seq tracks were visualized using Integrative Genomics Viewer (v2.3.77). Analyses were performed and plots generated using R (v3.2.2 or v3.4.3, packages ggplot2 (v2.2.1) and corrplot (v0.84)), SPICE 6.0 and GraphPad Prism (v7.0b). Except where otherwise specified, p-values < 0.05 were considered statistically significant. Simple comparisons were performed using parametric or non-parametric tests as appropriate and are described alongside the results.

### Study Approval

All participants have given informed consent for their participation in these studies. Individuals for inclusion in this analysis were selected at random and based on sample availability.

Recruitment for the HEATHER cohort was approved by the West Midlands—South Birmingham Research Ethics Committee (reference 14/WM/1104).

The SPARTAC trial was approved by the following authorities: the Medicines and Healthcare products Regulatory Agency (UK), the Ministry of Health (Brazil), the Irish Medicines Board (Ireland), the Medicines Control Council (South Africa) and the Uganda National Council for Science and Technology (Uganda). It was also approved by the following ethics committees in the participating countries: the Central London Research Ethics Committee (UK), Hospital Universitário Clementino Fraga Filho Ethics in Research Committee (Brazil), the Clinical Research and Ethics Committee of Hospital Clinic in the province of Barcelona (Spain), the Adelaide and Meath Hospital Research Ethics Committee (Ireland), the University of Witwatersrand Human Research Ethics Committee, the University of Kwazulu-Natal Research Ethics Committee and the University of Cape Town Research Ethics Committee (South Africa), Uganda Virus Research Institute Science and Ethics Committee (Uganda), the Prince Charles Hospital Human Research Ethics Committee and St Vincent’s Hospital Human Research Ethics Committee (Australia) and the National Institute for Infectious Diseases Lazzaro Spallanzani, Institute Hospital and the Medical Research Ethics Committee, and the ethical committee of the Central Foundation of San Raffaele, MonteTabor (Italy).

## Data Availability Statement

The raw data supporting the conclusions of this article will be made available by the authors, without undue reservation.

## Ethics Statement

The patients/participants provided their written informed consent to participate in this study. The details of the bodies responsible for approval of these studies are listed in “Study approval”.

## Author Contributions

GM, DS, WH, and JFr devised the study. GM, DS, NR, NO, and HB performed laboratory experiments. MP, EA and CW provided technical assistance with experimental design. GM and DS analyzed data and prepared figures for presentation. GM and JFr wrote the manuscript with critical input from all authors. JM managed the patient cohorts and collated demographic data. GM, MP, NR, JM, JT, MJ, AO, LP, NO, PZ, and HB processed clinical samples for the HEATHER cohort. JT, NN, JFo, and SF managed clinical sites and recruitment. NN, JFo, SF, CW, WH, and JFr led on study design and management. All authors contributed to the article and approved the submitted version.

## Funding

This work was supported by the Medical Research Council [grant no. MR/L006588/1] to JFr and the National Institute of Health Research Oxford Biomedical Research Centre.

## Conflict of Interest

WH is an employee of Merck and Company and holds equity in Tango Therapeutics and Arsenal Biosciences.

The remaining authors declare that the research was conducted in the absence of any commercial or financial relationships that could be construed as a potential conflict of interest.
