# Supplementary material for: Epigenetic Features of HIV-Induced T-Cell Exhaustion Persist Despite Early Antiretroviral Therapy
Source: Front Immunol. 2021 Jun 4;12:647688. doi: 10.3389/fimmu.2021.647688 (PMC8213372; doi:10.3389/fimmu.2021.647688)
Supplement: Supplementary file 1 [file DataSheet_1.pdf]

Fig. S1 – Gating strategy for flow cytometric and sorting experiments

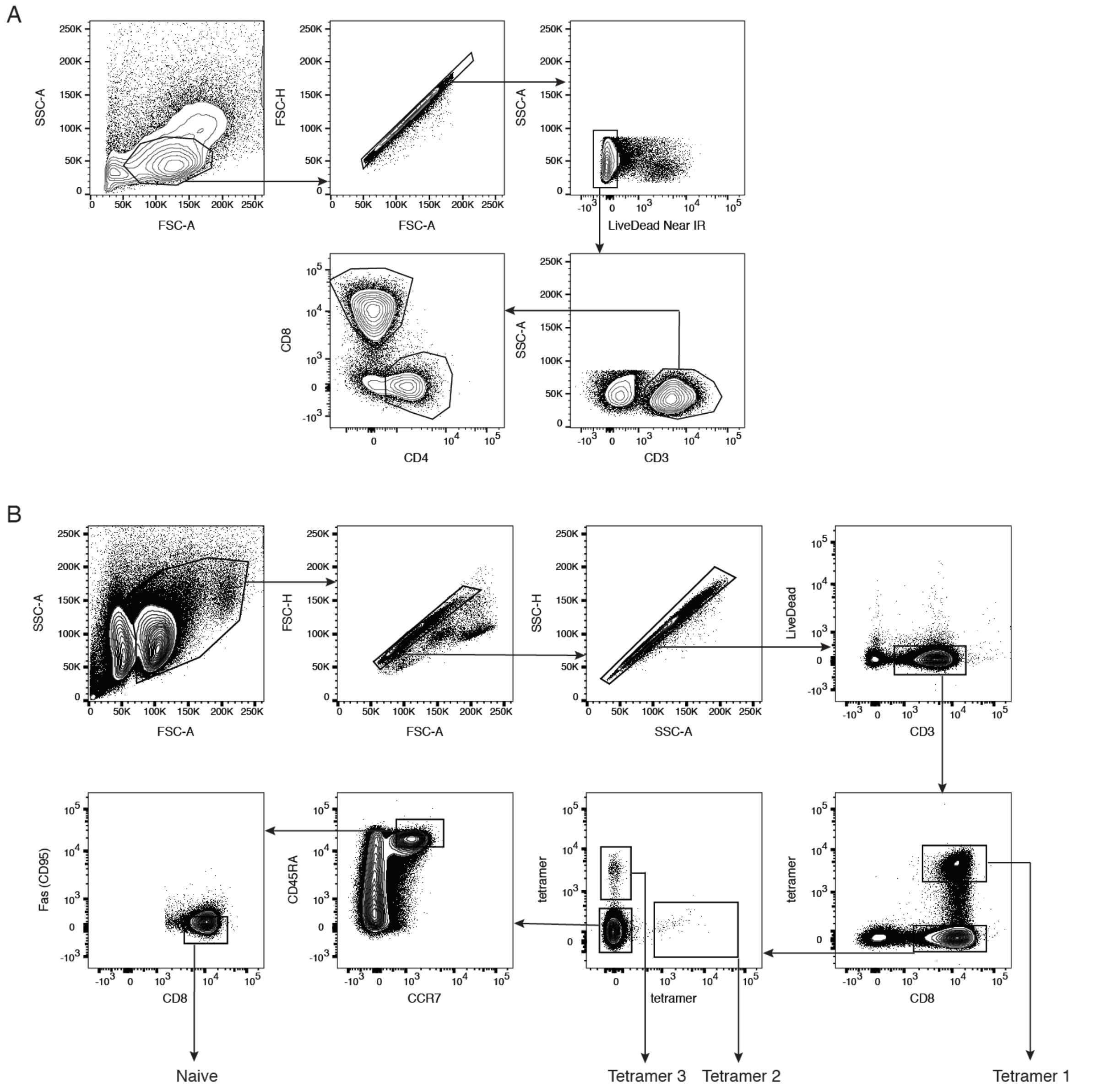

(A) Representative gating of CD4 and CD8 T cells for phenotyping experiments. Cells were gated based on FSC-A vs SSC-A characteristics. Following doublet and dead cell exclusion, CD3 T cells were gated against SSC-A. CD4 and CD8 T cells were then gated based on the reciprocal expression of these markers. (B) Representative gating of CD8 T cell populations for sorting experiments. Cells were gated based on FSC-A vs SSC-A characteristics. Following doublet exclusion, live CD3 T cells were gated. Tetramer-specific populations and bulk naïve cells were then sorted as shown.

Fig. S2 – PD-1 expression on CD8 T cell subsets defined by T-bet and Eomes expression

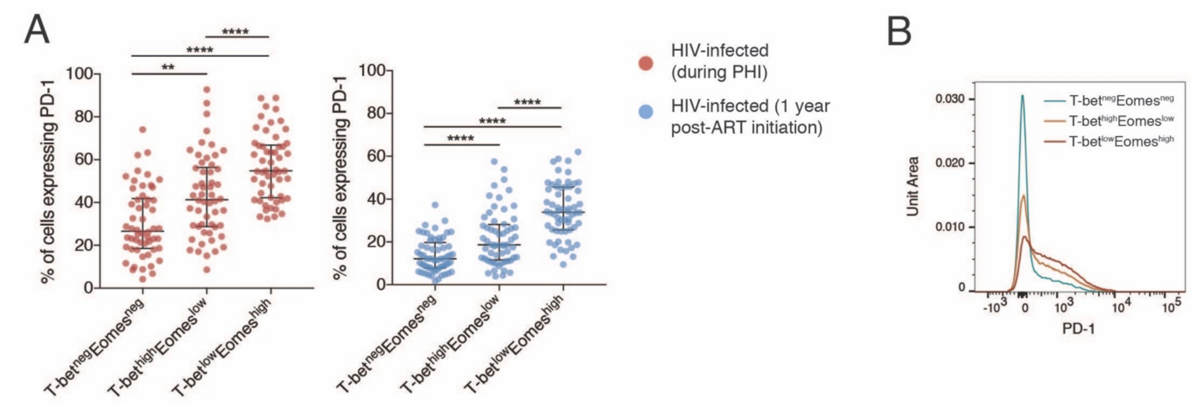

(A) The percentage of each subset (T-bet^{neg}Eomes^{neg}, T-bet^{high}Eomes^{low} and T-bet^{low}Eomes^{high}) expressing PD-1 is shown during PHI (red, left panel, n=54) and following 1 year of ART (blue, right panel, n=60). Groups were compared using a Friedman test (overall p for all tests <0.001) with subsequent pairwise comparisons performed with Dunn's test. Bars indicate median and interquartile range. ** indicates p<0.01; **** indicates p<0.0001. (B) Representative flow plots from each subset are overlaid from one donor during PHI.

Fig. S3 – Immune checkpoint receptor expression on CD8 T cell memory subsets during primary HIV infection

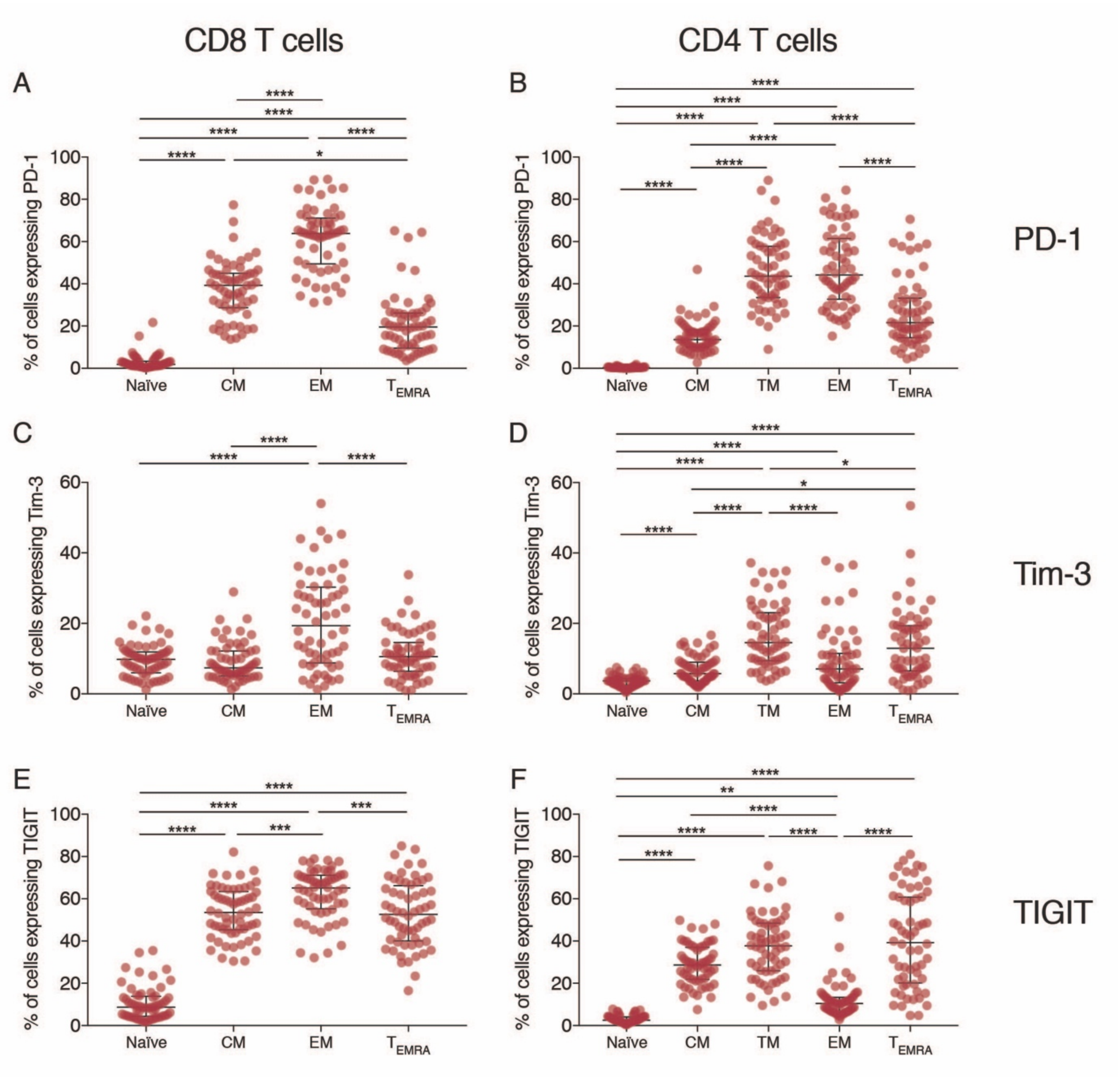

The expression of PD-1 (A-B), Tim-3 (C-D) and TIGIT (E-F) on naïve, central memory (CM), transitional memory (TM), effector memory (EM) and T_{EMRA} cells is shown for HIV-infected individuals during PHI (n=60). Panels A, C, and E show ICR expression on CD8 T cells and panels B, D, and F show the same on CD4 T cells. Groups were compared using a Friedman test (overall $p<0.05$ for all cases), with subsequent pairwise comparison with Dunn's test corrected for multiple comparisons. Bars indicate median and interquartile range. * indicates $p<0.05$; ** indicates $p<0.01$; *** indicates $p<0.01$; **** indicates $p<0.001$.

Fig. S4 – Increases in immune checkpoint receptor expression during primary HIV infection

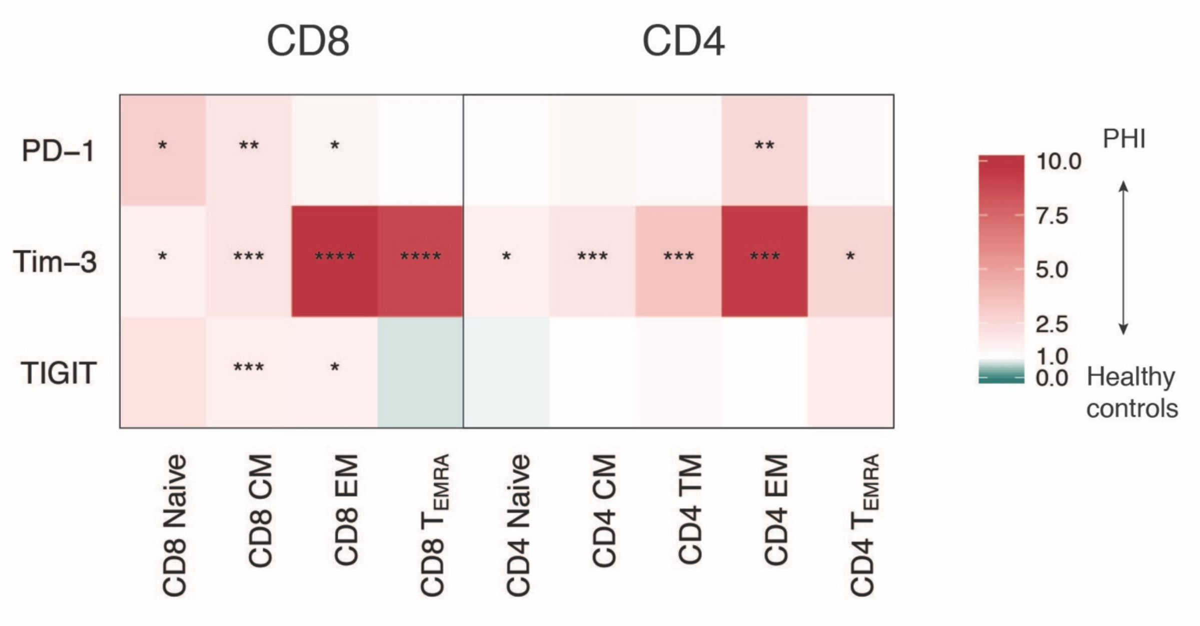

Increases in ICR expression during PHI. Summary of comparisons of ICR expression between healthy controls (n=10) and individuals during PHI (n=60). Blocks, each corresponding to an ICR measured on a CD4 or CD8 T cell subset, are shaded according to the fold change in PHI relative to healthy controls. Groups were compared using Mann-Whitney tests and adjustment of p-values for multiple comparisons was performed across all comparisons presented here to control the false discovery rate. Asterisks and listed p-values correspond to the adjusted p-value. * indicates p<0.05; ** indicates p<0.01; *** indicates p<0.001; **** indicates p<0.0001.

Fig. S5 – Viral load and sampling timing for individuals used for epigenetic characterisation

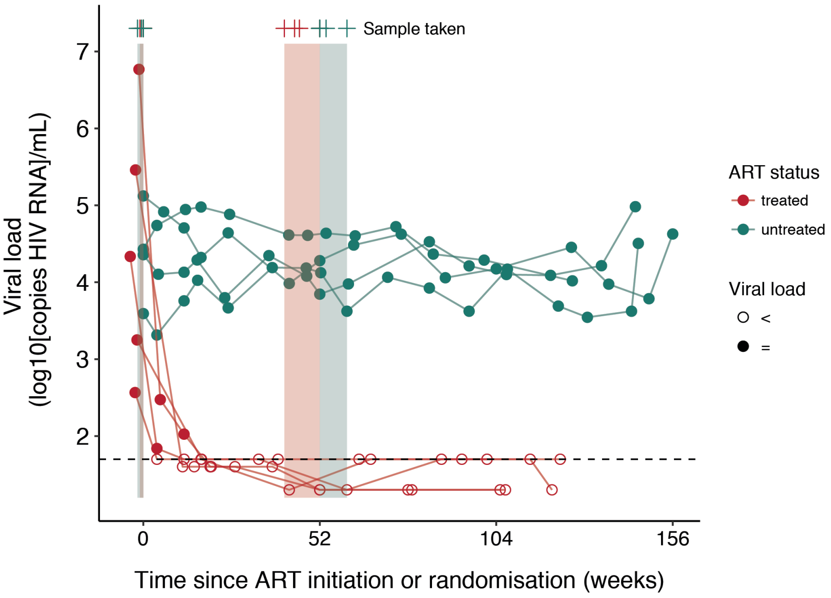

Viral load dynamics and sampling timing for individuals used for epigenetic characterisation are shown (n=9). Individuals who received ART are shown in red, and those that were untreated are shown in green. VL measurements below the limit of detection are shown as open circles. Shading indicates the window during which samples were taken, with the individual timing indicated at the top.

Table S1 - Demographic and clinical characteristics of participants

Sex	
• Male	66 (100%)
Age	34 [28 - 41]
Time between date of confirmed HIV+ test and ART initiation (days)	29 [14 - 45]
Time between estimated date of seroconversion and ART initiation (days)	52 [34 - 98]
Time between ART initiation and first VL <50 copies/ml (days)[1]	133 [87 - 238]
Baseline CD4 T cell count (cells/µL)[2]	530 [406 - 652]
Baseline CD8 T cell count (cells/µL)[2]	1028 [825 - 1334]
Baseline CD4 to CD8 ratio[2]	0.52 [0.36 – 0.79]
Baseline viral load (log_{10} copies[HIV RNA]/ml)	5.4 [4.4 – 6.4]
Method for diagnosing primary HIV infection	
• Antigen positive (p24 or PCR) but antibody negative	26 (39%)
• Rising antibody titre	1 (2%)
• Negative test within 6 months of positive test	31 (47%)
• Recent incidence testing algorithm	8 (12%)
Mode of acquisition	
• MSM	59 (89%)
• MSW	1 (2%)
• Unknown/unrecorded	6 (9%)
Initial ART regimen	
Backbone	
• tenofovir containing	58 (88%)
• abacavir containing	5 (8%)
• unknown/unrecorded	3 (5%)
Additional agent(s)	
• protease inhibitor	37 (56%)
• NNRTI	12 (18%)
• integrase inhibitor	13 (20%)
• protease inhibitor + integrase inhibitor	1 (2%)
• unknown/unrecorded	3 (5%)

Demographic and baseline clinical characteristics of included participants from the HEATHER cohort. Values given represent n (%) for categorical variables and median (interquartile range) for continuous variables. Abbreviations: MSM, men who have sex with men; MSW, men who have sex with women, NNRTI, non-nucleoside reverse-transcriptase inhibitor.

1 63/66 individuals were virologically suppressed to <50 copies/ml by 1 year visit

2 Data available for 65/66 individuals

Table S2 – Variables included in principle component analysis presented in Figure 3A

Parameters
CD4 Naïve %
CD4 CM %
CD4 TM %
CD4 EM %
CD4 T_{EMRA} %
CD8 Naïve %
CD8 CM %
CD8 EM %
CD8 T_{EMRA} %
CD4 Naïve PD-1 %
CD4 CM PD-1 %
CD4 TM PD-1 %
CD4 EM PD-1 %
CD4 T_{EMRA} PD-1 %
CD4 Naïve Tim-3 %
CD4 CM Tim-3 %
CD4 TM Tim-3 %
CD4 EM Tim-3 %
CD4 T_{EMRA} Tim-3 %
CD4 Naïve TIGIT %
CD4 CM TIGIT %
CD4 TM TIGIT %
CD4 EM TIGIT %
CD4 T_{EMRA} TIGIT %
CD8 Naïve PD-1 %
CD8 CM PD-1 %
CD8 EM PD-1 %
CD8 T_{EMRA} PD-1 %
CD8 Naïve Tim-3 %
CD8 CM Tim-3 %
CD8 EM Tim-3 %
CD8 T_{EMRA} Tim-3%
CD8 Naïve TIGIT %
CD8 CM TIGIT %
CD8 EM TIGIT %
CD8 T_{EMRA} TIGIT %
CD8 T-bet^{high}Eomes^{low} %
CD8 T-bet^{low}Eomes^{high} %
CD8 T-bet^{neg}Eomes^{neg} %
CD4 CD38 %
CD8 CD38 %

Table S3 - Demographic and clinical characteristics of participants used for sorting experiments

	**Untreated (SPARTAC)**	**Treated (HEATHER)**	**p-value**
Sex			
• *Male*	4 (100%)	5 (100%)	
Age (years)	36 [27-42]	34 [33-41]	1
Viral subtype			
• *B*	4 (100%)	4 (80%)	
• *CRF02-AG*	-	1 (20%)	
Country of recruitment			
• *United Kingdom*	2 (50%)	5 (100%)	
• *Australia*	2 (50%)	-	
Time between estimated date of seroconversion and baseline visit (days)	38 [31 – 139]	32 [16 – 165]	0.71
Time between ART initiation and first VL <50 copies/mL (days)	N/A	84 [28 – 138]	
Baseline HIV RNA (log_{10} copies/mL)	4.4 [3.4 – 5.8]	4.3 [2.6 – 6.8]	0.73
Baseline CD4 T cell count (cells/µL; median)	900 [428.5 – 1190]	652 [539 – 1200]	1
Baseline CD4:CD8 ratio (median [range])	0.59 [0.32 – 0.65]	0.80 [0.36 - 1.28]	0.41

Demographic and baseline clinical characteristics of included participants from the HEATHER and SPARTAC cohorts. Values given represent n (%) for categorical variables and median (IQR range) for continuous variables. Where relevant, groups were compared using a Mann-Whitney U test.

Table S4 – Tetramers used for sorting experiments and contemporaneous plasma viral sequences

Patient ID	Tetramer used	Gene	Subtype	Sequence at locus - baseline	Sequence at locus – 1 year	Tetramer population still seeing virus	Retained in analysis
SAQ038001	A*03:01 RLRPGGKKK	Gag	B	RLRPGGKKK	No sample	Yes	Yes
SAX032001	A*03:01 RLRPGGKKK	Gag	B	RLRPGGKK[**R**/K]	RLRPGGKK[**R**/K]	Yes [A]	Yes
	B*07:02 GPGHKARVL	Gag		GPGHKAR**I**L	GPGHKAR[V/**I**]L	Yes [B]	Yes
SUK036043	B*57:01 KAFSPEVIPMF	Gag	B	KAFSPEVIPMF	KAFSPEVIPMF	Yes	Yes
	B*57:01 TSTLQEQIAW	Gag		TS**N**LQEQIAW	TS**N**LQEQIAW	No [C]	No
SUW036083	B*53:01 YPLTFGWCF	Nef	B	YPLTFGWCF	YPLTFGWCF	Yes	Yes
CW001	B*07:02 GPGHKARVL	Gag	CRF02-AG	GP[G/**S**]HKARVL	-	Yes [D]	Yes
CW006	B*07:02 GPGHKARVL	Gag	B	GP**A**HKARVL	-	Yes [E]	Yes
ST001	B*57:01 TSTLQEQIAW	Gag	B	TSTLQEQI**G**W	-	Yes [F]	Yes
ST003	B*57:01 KAFSPEVIPMF	Gag	B	KAFSPEVIPMF	-	Yes	Yes
	B*57:01 TSTLQEQIAW	Gag		TS**N**LQEQI**Q**W	-	No [C]	No
ST013	A*03:01 RLRPGGKKK	Gag	B	RLRPGG[K/**R**]KK	-	Yes [G]	Yes
	B*07:02 GPGHKARVL	Gag		GP[G/**S**]HKA[R/**K**]VL	-	Yes [D]	Yes

A - RLRPGGKK**R** is an escape mutation which is poorly recognised by T cells which respond to RLRPGGKKK during primary HIV infection[1]. CD8 T cells which respond to RLRPGGKKK show decreased avidity, improved functionality and decreased PD-1 expression[2]. In this individual the wild type RLRPGGKKK is present as a variant at both time point, and there is ELISpot evidence of robust T cell response to this epitope.

B - GPGHKAR**I**L is not a described escape mutation and there is ELISpot evidence of T cell responses to this epitope.

C - TS**N**LQEQIAW is a well-characterised early escape mutation which is not cross-recognised by wild-type T cells[3]. Similarly, TS**N**LQEQI**Q**W is also escaped, with the Q at position 9 acting as compensatory mutation for the fitness cost associated with position 3 N substitution[4].

[1] Du VY, Bansal A, Carlson J, Salazar-Gonzalez JF, Salazar MG, Ladell K, et al. HIV-1-Specific CD8 T Cells Exhibit Limited Cross-Reactivity during Acute Infection. J Immunol. 2016;196(8):3276-86.
[2] Streeck H, Brumme ZL, Anastario M, Cohen KW, Jolin JS, Meier A, et al. Antigen Load and Viral Sequence Diversification Determine the Functional Profile of HIV-1–Specific CD8+ T Cells. *PLOS Medicine.* 2008;5(5):e100.
[3] Leslie AJ, Pfafferott KJ, Chetty P, Draenert R, Addo MM, Feeney M, et al. HIV evolution: CTL escape mutation and reversion after transmission. *Nature medicine.* 2004;10(3):282-9.
[4] Tang Y, Huang S, Dunkley-Thompson J, Steel-Duncan JC, Ryland EG, St John MA, et al. Correlates of spontaneous viral control among long-term survivors of perinatal HIV-1 infection expressing human leukocyte antigen-B57. *AIDS.* 2010;24(10):1425-35.

D - GP**S**HKARVL is not an escape mutation but a better binding variant[5].

E - GP**A**HKARVL variant is not a described escape mutation.

F - TSTLQEQI**G**W is the B clade consensus sequence and not a described escape mutation.

G - RLRPGG**R**KK is described as having similar binding affinity to RLRPGGKKK when tested experimentally[6], and is likely to be cross-recognised.

---

[5] Kloverpris HN, Adland E, Koyanagi M, Stryhn A, Harndahl M, Matthews PC, et al. HIV subtype influences HLA-B*07:02-associated HIV disease outcome. *AIDS Res Hum Retroviruses.* 2014;30(5):468-75.

[6] Milicic A, Edwards CT, Hue S, Fox J, Brown H, Pillay T, et al. Sexual transmission of single human immunodeficiency virus type 1 virions encoding highly polymorphic multisite cytotoxic T-lymphocyte escape variants. *J Virol.* 2005;79(22):13953-62.

Table S5 - Primers used for sequencing of gag and nef sequences from plasma virus

Primer	*Gene*	*Sequence*	*Position in HXB2*
**cDNA synthesis and first-round amplification**			
gagC5OP (forward)	*gag*	5'-CTCTAGCAGTGGCGCCCGAA-3'	627-646
gagC3OP (reverse)	*gag*	5'-GCTGTCATCTCTTGTGGGCTGT-3'	2023-2045
nef5OP (forward)	*nef*	5'-TTCCAGTCAGACCTCAGGTAC-3'	8999-9019
nef3OP (reverse)	*nef*	5'-TCTAGTTACCAGAGTCACACAA-3'	9656-9677
**Second-round amplification and sequencing reaction**			
gag5IP (forward)	*gag*	5'-ACTCGGCTTGCTGAAGTGC-3'	696-714
gag3IP (reverse)	*gag*	5'-CAATTTCTGGCTATGTGCCC-3'	1984-2003
gag3F (forward)	*gag*	5'-GGTCAGCCAAAATTACCCTATAG-3'	1170-1192
gag2R (reverse)	*gag*	5'-TCTGCAGCTTCCTCATTGATGGTC-3'	1398-1421
gag1R (reverse)	*gag*	5'-ACCGACGCTCTCGCACCCATC-3'	789-809
gag5F (forward)	*gag*	5'-GGAACAAATAGCATGGATGAC-3'	1521-1541
gagF2nst (forward)	*gag*	5'-GCGGAGGCTAGAAGGAGAGAGATGG-3'	769-793
gagF1b2 (reverse)	*gag*	5'-CTGCACTATAGGATAATTTTGAC-3'	1175-1197
gagF1b3 (forward)	*gag*	5'-GACACCAAGGAAGCCTTAG-3'	1075-1093
gagF1b4 (reverse)	*gag*	5'-CTCCCACTGGAACAGGTG-3'	1550-1567
gagF1b5 (forward)	*gag*	5'-GGAACAAATAGCATGGATGAC-3'	1521-1541
nef5IP (forward)	*nef*	5'-CTTTTTAAAAGAAAAGGGGGGAC-3'	9063-9085
nef3IP (reverse)	*nef*	5'-TCAGATCTGGTCTAACCAGAG-3'	9545-9565

The binding position is given relative to a subtype B reference viral genome (HXB2). Abbreviations: cDNA, complementary DNA.
